# Tenovin-1 Ameliorates Renal Fibrosis in High-Fat-Diet-Induced Diabetic Nephropathy via Antioxidant and Anti-Inflammatory Pathways

**DOI:** 10.3390/antiox11091812

**Published:** 2022-09-14

**Authors:** Amit Kundu, Sreevarsha Gali, Swati Sharma, Jae Hyeon Park, So Young Kyung, Sam Kacew, In Su Kim, Kwang Youl Lee, Hyung Sik Kim

**Affiliations:** 1 School of Pharmacy, Sungkyunkwan University, 2066, Seobu-ro, Jangan-gu, Suwon 440-746, Korea; 2 McLaughlin Centre for Population Health Risk Assessment, University of Ottawa, Ottawa, ON K1N 6N5, Canada; 3 College of Pharmacy, Chonnam National University, Yongbong-ro, Buk-gu, Gwangju 61186, Korea

**Keywords:** high-fat diet, diabetic nephropathy, Tenovin-1, oxidative stress, renal fibrosis

## Abstract

High-fat diet (HFD)-induced obesity has been involved in the development of diabetic nephropathy (DN). Tenovin-1, a potent selective SIRT1/2 inhibitor, regulates various target proteins. The present study evaluated the protective effect of Tenovin-1 against renal fibrosis in HFD-induced Zucker diabetic fatty (ZDF) rats. Rats were fed a normal chow diet or HFD. Tenovin-1 (45 mg/kg) administered to HFD-fed rats decreased inflammatory cytokine expression in the serum of the rats. The antioxidant status and oxidative damage to lipids or DNA were significantly restored by Tenovin-1. Additionally, Tenovin-1 reduced the levels of blood urea nitrogen (BUN), serum creatinine (sCr), microalbumin, and urinary protein-based biomarkers in the urine of HFD-fed rats. The abnormal architecture of the kidney and pancreas was restored by Tenovin-1 administration. Tenovin-1 also reduced apoptosis in the kidneys of the HFD-fed rats and HG-treated NRK-52E cells. It significantly lowered the levels of ECM proteins in the kidneys of HFD-fed rats and HG-treated NRK-52E cells. Additionally, Tenovin-1 markedly reduced claudin-1, SIRT1, and SIRT2, but increased SIRT3 and SIRT4 in HFD-fed rats and NRK-52E cells treated with HG. Furthermore, Tenovin-1 altered epidermal growth factor receptor (EGFR), platelet-derived growth factor receptor-β (PDGFR-β), and signal transducer and activator of transcription 3 (STAT3) levels in the kidneys of HFD-fed rats. Conclusively, this study shows that Tenovin-1 can be a potential candidate drug for the treatment of HFD-induced renal fibrosis, in vivo and in vitro models.

## 1. Introduction

Diabetic nephropathy (DN) is a major microvascular disorder associated with diabetes mellitus (DM). DM is an emerging global health burden, and it is projected that approximately 439 million people will suffer from DM by 2030 [1]. Alterations in the metabolism of protein, glucose, and fat are characteristic features of DM, a progressive metabolic disorder. Changes in metabolism due to DM can result in major microvascular disorders, such as nephropathy, neuropathy, and retinopathy [2,3].

Poor glycemic control and the buildup of advanced glycation end products (AGEs), which cause tissue degeneration in association with severe kidney impairment, are the underlying causes of DN [4]. The progression of DN and related complications are associated with numerous pathophysiological processes, such as oxidative stress, chronic inflammation, and apoptosis [5]. The major pathological characteristics of DN include glomerulosclerosis, which is due to the upregulation of the expression of extracellular matrix (ECM) proteins, such as collagen IV, α-smooth muscle actin (α-SMA), and fibronectin in mesangial cells, and renal interstitial fibrosis. DN is involved in mediation of the epithelial–mesenchymal transition, which is characterized by the loss of epithelial proteins and the acquisition of the mesenchymal protein α-SMA [6].

Basement membrane thickening, mesangial cell expansion, podocyte loss within the glomeruli, urinary albumin leakage, and alteration of the capacity of glomerular function are key clinical features of patients with DN [7,8]. The impairment of renal activity due to mesangial expansion, tubulointerstitial fibrosis, and irreversible deterioration is associated with glomerular ECM accumulation, which enhances the production of matrix proteins and ultimately leads to characteristic morphological transformations in diabetic kidneys [7,9].

Numerous SIRT1 inhibitors have demonstrated their therapeutic efficacy in different disease models. Among them, Tenovin-1 is reported to inhibit the activity of SIRT1/2 [10]. Tenovin-1 (N-[[[4-(acetylamino) phenyl] amino] thioxomethyl-4-(1,1-dimethyl ethyl)] benzamide) is an inhibitor of the deacetylase activity of SIRT1 and SIRT2 [11]. The inhibition of HDAC activity by Tenovin-1 has shown promising results in human melanoma, where it exhibited a better inhibition of cell proliferation and viability when compared with Sirtinol and Ex-527 [12,13]. However, the role of Tenovin-1 in fibrogenesis remains undetermined. A recently published report revealed that sirtinol (a selective SIRT1/2 inhibitor), EX-527 (SIRT1 inhibitor), and AGK2 (SIRT2 inhibitor) suppressed interstitial fibroblast stimulation and reduced interstitial fibrosis in both cultured renal interstitial fibroblast cells (NRK-49F) and a high-fat diet (HFD)-induced ZDF rat model [14,15]. Such selective SIRT inhibitors also reduce the expression of fibroblast activation markers including collagen I, α-SMA, and fibronectin in dose-dependent manners [15,16]. Treatment with sirtinol attenuated the expression of α-SMA, fibronectin, and collagen I in the damaged kidneys of a nephropathy mouse model. In particular, SIRT1/2 inhibitors may contribute to therapeutic efficacy for the management of diabetic kidney disorders [15].

We hypothesized that inhibiting SIRT1/2 by Tenovin-1 reduces the blood glucose levels, and reducing high-fat-diet-induced renal interstitial fibroblast activation and renal fibrosis by suppressing the phosphorylation of growth factor receptors, EGFR, and PDGFR, and the consequent inactivation of the STAT3 signaling inhibits the expression of inflammatory mediators and oxidative stress markers. Therefore, the beneficial effect of Tenovin-1 may be mediated primarily by its ability to block the activation of growth factor receptors and pharmacologic blockade of the STAT3 signaling pathway.

Based on this hypothesis, we explored the effects of Tenovin-1 on renal fibrosis in HFD-induced ZDF rats. In the HFD-induced rat model in the present study, we observed significant renal damage, changes in biochemical parameters, and renal histopathological injury. The present study investigated whether an SIRT1/2 inhibitor, Tenovin-1, has a protective function against HFD-induced renal fibrosis and its underlying mechanisms.

## 2. Materials and Method

### 2.1. Chemicals and Materials

Tenovin-1 was purchased from Cayman Chemical (Ann Arbor, MI, USA). 3-indoxyl sulfate (3-IS) was procured from Sigma-Aldrich (St. Louis, MO, USA). The OxiSelectTM AGEs Competitive ELISA Kit was obtained from Cell Biolabs (San Diego, CA, USA). The malondialdehyde (MDA) assay kit (TBARS assay kit) was obtained from Cayman Chemicals Company, Ann Arbor, MI, USA. Catalase (CAT) and SOD activities were investigated using Ellman’s reagent-based calorimetric assay kits, 8-hydroxy-2-deoxyguanosine (8-OHdG), and the Hydroxyproline Assay Kit from Cell Bio Labs. TGF-β, interleukin 1 beta (IL-1β), interleukin-6 (IL-6), and interleukin-10 (IL-10) ELISA kits were obtained from Abcam, Cambridge, MA, USA. The primary antibodies specific to neutrophil gelatinase-associated lipocalin (NGAL), kidney injury molecule-1 (Kim-1), selenium binding protein (SBP-1), E-cadherin, collagen-1, fibronectin, TGF-β, α-SMA, vimentin, α-tubulin, SIRT1, SIRT2, SIRT3, SIRT4, claudin-1, p-EGFR, EGFR, p-PDGFRβ, PDGFRβ, p-STAT3, STAT3, and β-actin were purchased from Cell Signaling Technology (Danvers, MA, USA). Horseradish peroxidase (HRP)-conjugated secondary antibodies were purchased from Santa Cruz Biotechnology (Santa Cruz, CA, USA). DMEM, fetal bovine serum (FBS; cat. no. 10099-141), and trypsin (cat. no. 25300-054) were obtained from Gibco Invitrogen Corporation (Carlsbad, CA, USA). An FITC Annexin V apoptosis detection kit (cat. no. 556547) was purchased from BD Pharmingen. 4,6-diamidino-2-phenylindole dihydrochloride (DAPI; cat. no. D9542) was obtained from Sigma-Aldrich.

### 2.2. Experimental Design

Male ZDF rats (adult, body weight, 250 ± 25 g) were provided by Central Lab Animal Inc. (Seoul, Korea). All animals were housed in a specific pathogen-free room with an automatically regulated temperature (25 ± 0.5 °C), with a relative humidity of 53–57% and a 12 h light/dark cycle. The rats were housed for seven days before beginning the experiments for acclimatization. The study methodology was approved by the Animal Ethics Committee Recommendations (SKKUIACUC2018-10-32-1). The care and use of animals were in accordance with all applicable international, national, and institutional standards. All procedures that involved animals were performed in accordance with the standard ethical rules of the institution where the studies took place.

**Figure 1:**
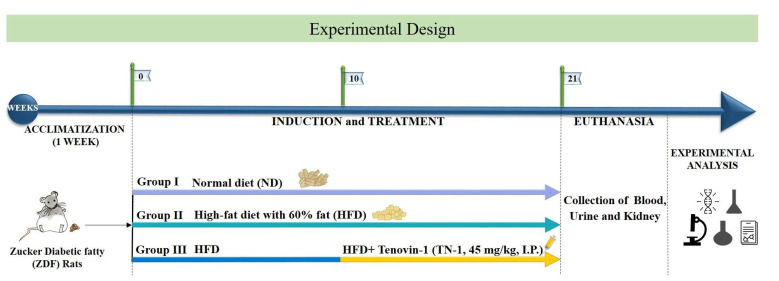
Experimental design. After one week of acclimatization, ZDF rats were randomly divided into 2 groups: the group that received a standard chow diet was the normal diet group (ND, *n* = 6), and the group that received a high-fat diet was the experimental group (HFD, *n* = 12). The rats were split into two groups after 10 weeks of HFD feeding (*n* = 6 in each group), with one group receiving only an HFD (*n* = 6) and the other receiving an HFD plus Tenovin-1 treatment (HFD + Tenovin-1) for 10 weeks. ND, normal diet; HFD, high-fat diet.

The high-fat diet (HFD), comprising carbohydrates, proteins, minerals, fiber, and vitamins with 60% fat, was procured from Research Diets Inc (New Brunswick, NJ 08901, USA). Diabetes was induced by feeding the rats an HFD for 10 weeks. Tenovin-1 (45 mg/kg body weight) was injected intraperitoneally (i.p.) for ten weeks to the HFD-fed rats [11]. The control group received a normal cholesterol-free diet. Blood collection was performed from the tail vein in the morning, and blood glucose levels were estimated using a glucometer (ACCU-CHEK; Daeil Pharm. Co, Ltd., Seoul, Korea). Body weight and blood glucose levels were measured weekly. Glucose concentrations greater than 250 mg/dL in rats indicated diabetes, and these rats were used for further experiments. The HFD-induced diabetic rats were randomly divided into two groups, with six rats in each group (*n* = 6). Blood samples were collected at the beginning and at the end of the experiment. Blood samples were centrifuged at 2000× *g* for 10 min to collect the serum. Urine samples were collected weekly. Metabolic cages were used for urine collection. Serum and urine samples were stored at −20 °C until the completion of the experiments. Rat kidneys, livers, and pancreases were perfused with saline after sacrifice to remove blood stains and placed in −80 °C conditions until use (Figure 1).

### 2.3. Analysis of Serum Biochemical Parameters

The serum samples obtained from the blood of the experimental animals were stored in sterile tubes at −80 °C until analysis. Blood urea nitrogen (BUN) and serum creatinine (SCr) concentrations were analyzed using a Vet Scan analyzer (Abaxis, Inc., Union City, CA, USA).

The OxiSelectTM AGEs Competitive ELISA Kit from Cell Biolabs (San Diego, CA, USA) was used to determine the concentrations of AGEs in the serum. The circulating AGEs were analyzed depending on the sample’s fluorescence or absorbance in each well, as measured using a fluorescence microplate reader (Molecular Devices, San Jose, CA, USA) at 450 nm.

### 2.4. Analysis of Urinary Parameters

Rats were placed in individual metabolic cages for 24 h while fasting and given unrestricted access to water. Rat urine was collected under cool conditions. To remove cellular debris and other insoluble substances, the urine was immediately centrifuged at 879× *g* and 4 °C for 10 min. The urine supernatant was aliquoted into sterile tubes and stored at −80 °C until use. The urinary concentrations of creatinine (Cr) and microalbumin were analyzed using a Hitachi 7180 autoanalyzer (Hitachi, Tokyo, Japan).

### 2.5. Assessment of Oxidative Stress

An ice-cold phosphate buffer (pH 7.4) was used to homogenize frozen kidney tissues (25 mg). The homogenized tissues were centrifuged for 10 min at 1500× *g*. The obtained supernatant was collected in a separate tube. Further assessment was performed using the supernatant. The malondialdehyde (MDA) content was estimated using a thiobarbituric acid reactive substances assay kit (TBARS assay kit, Cayman Chemicals Company, Ann Arbor, MI, USA). In accordance with the manufacturer’s instructions, the colorimetric assay kit (MDA-586; OxisResearChTM, Portland, OR, USA) was used to measure the activity of superoxide dismutase (SOD). Protein concentrations were quantified using a standard method [17]. MDA contents and catalase (CAT) and SOD activities in kidney tissues were investigated using Ellman’s reagent-based calorimetric assay with commercially available assay kits. Additionally, using an ELISA kit, the amount of 8-hydroxy-2-deoxyguanosine (8-OHdG), a byproduct of oxidative DNA damage, was determined in urine (Cell Bio Labs). The concentrations of reduced glutathione (GSH) in the kidney tissue were measured using Ellman’s reagent-based assay with commercially available kits (Cayman Chemicals Company, Ann Arbor, MI, USA).

### 2.6. Assessment of Diabetic Renal Inflammation

In accordance with the manufacturer’s instructions, an ELISA assay kit (Abcam, Cambridge, MA, USA) was used to measure the effect of Tenovin-1 on the NF-κB pathway and find the concentrations of inflammatory cytokine mediators. The concentrations of the major cytokine factors, such as TGF-β, interleukin-6 (IL-6), interleukin-10 (IL-10), and interleukin 1 beta (IL-1β), were evaluated using standard curves.

### 2.7. Western Blot Analysis

The expressions of proteins in kidney tissues, including SIRT1 (1:1000), SIRT2 (1:1000), SIRT3 (1:1000), SIRT4 (1:1000), and claudin-1 (1:1000), were measured. The fibrous protein markers in renal cortex including E-cadherin (1:1000), α-SMA (1:500), collagen-1 (1:500), vimentin (1:1000), α-tubulin (1:1000), TGF-β (1:1000), fibronectin (1:500) EGFR, p-EGFR (1:1000), PDGFRβ (1:1000), p-PDGFRβ (1:1000), STAT3 (1:1000), p-STAT3 (1:1000), Bcl-2 (1:1000), Bax (1:1000), and cleaved caspase-3 (1:1000), were analyzed using Western blotting. The expressions of urinary protein markers, such as KIM-1 (1:1000), NGAL (1:1000), SBP (1:1000), and vimentin (1:500), were also analyzed. For the experiment, the RIPA lysis buffer was used to homogenize the cortex of the frozen kidney tissues (around 50 mg). Homogenates were transferred to ice for 30 min and cold-centrifuged at 4 °C and 12,000× *g* for 15 min. The protein concentrations in the supernatants were estimated using the BCA protein assay (Thermo Fisher Scientific, Waltham, MA, USA)). Sodium dodecyl sulfate–polyacrylamide gel was used for the separation of protein samples (60–80 µg). A polyvinylidene fluoride (PVDF) membrane was used to transfer the protein from the gel, and PVDF membranes were treated overnight with primary antibodies at 4 °C after being blocked with a 5% milk buffer at room temperature. The PVDF membranes were then washed with Tris-buffered saline with 0.1% Tween 20 detergent (TBST) and incubated with secondary antibodies (1:10,000) at room temperature for 1 h. The ECL chemiluminescence reagent was used to quantify protein expression and band intensity.

### 2.8. Determination of 4-Hydroxyproline Concentration

Kidney tissues (100 mg) were incubated at 120 °C for 12 h for hydrolyzation and followed by homogenization with 10 mL 5 N HCl. A colorimetric assay kit (Hydroxyproline Assay Kit, Cell Biolabs) was used to measure the 4-hydroxyproline concentration according to the manufacturer’s instructions.

### 2.9. Analysis of the Concentration of 3-IS

The 3-IS content was estimated in the serum, urine, and kidney tissues using high-performance liquid chromatography (HPLC). The extraction of the samples was performed using 70% acetonitrile, an organic solvent, in a multi-step process. The extracted samples were mixed thoroughly with 2-naphthalene sulfonic acid used as an internal standard, and measured using HPLC (Gilson, LC-321322350) at 280 nm. The retention time of 3-IS was 6.8 min, and the extraction recovery was 84%. The HPLC apparatus used in the present study comprised a Gilson pump (LC-321322350 pump), an autosampler (Gilson-234), and a UV (UV/VIS-151) detector (Gilson, France). A C18 column from Agilent (Torrance, CA, USA) with a pre-column (250 mm 4.6 mm, and 5 m) was used for detection and quantification with a flow rate of 0.9 mL/min at room temperature. A 70:30 *v*/*v* combination of acetonitrile and 0.1% trifluoroacetic acid in Milli Q water was employed as the isocratic mobile phase.

### 2.10. Histopathological Examinations

The pancreas and kidneys tissues were immediately preserved in 10% buffer formaldehyde. To identify morphological changes in the tissues, paraffin slices of the tissues were produced for hematoxylin and eosin (H&E) staining. Masson’s trichrome (MT) staining using the Trichrome Gomori One-Step Aniline Blue Stain kit (Newcomer Supply, Middleton, WI, USA) was applied to examine the accumulation of collagen in the kidney tissues.

### 2.11. Immunohistochemistry and Immunofluorescence Analysis

Immunohistochemical analysis was conducted to investigate α-SMA, collagen-1, TGF-β, SIRT1, SIRT2, SIRT3, SIRT4, and claudin-1 expression. The slides were placed in a xylene chamber, followed by hydration in graded alcohol and water. The slides were moved to a chamber with 3% H_2_O_2_ to quench peroxidase activity. To prevent non-specific binding sites, the samples were treated in 4% bovine serum albumin for 1 h at 37 °C, followed by washing with TBS three times, and then incubation with primary antibodies (α-SMA 1:200, TGF-β 1:500, Collagen-1 1:1000, SIRT1 1:1000, SIRT2 1:1000, SIRT3 1:1000, SIRT4 1:1000, and claudin-1 1:1000) at 4 °C overnight. After incubating with specific antibodies, the membranes were washed three times with TBS, a secondary antibody conjugated with HRP was added, and the sections were incubated at room temperature for 45 min. The slides were counterstained with hematoxylin after immunostaining using diaminobenzidine tetrahydrochloride (DAB) as a visualizing agent.

Immunofluorescence staining was performed to investigate the levels of expression of α-SMA, collagen-1, and TGF-β. The slides were incubated with primary antibodies (α-SMA 1:500, TGF-β 1:200, and Collagen-1 1:200) at 4 °C overnight. After 3 washes with TBS, a secondary antibody labelled with FITC (fluorescein isothiocyanate) was added. DAPI was used to stain the nuclei. Histological investigation was performed to determine the alteration under a microscope (Zeiss Axiophot, Oberkochen, Germany) without knowledge of the identities of the various groups.

### 2.12. TUNEL Assay

To investigate apoptotic DNA fragmentation, a terminal deoxynucleotidyl transferase dUTP nick end labeling (TUNEL) assay was used. Diabetic rat liver tissue was examined using the DeadEndTM colorimetric technique (Promega, Madison, WI, USA) to identify apoptotic cells.

### 2.13. mRNA Expression Analyses

Total RNA was extracted (from the kidneys) using the TRIzol reagent (ref. no. 15596018, Life Technologies, Carlsbad, CA, USA). RNA was quantified using a spectrophotometer (NanoDrop Technologies Inc., Wilmington, DE, USA) at 260 and 280 nm, and approximately 2 µg of RNA was used, followed by cDNA synthesis using Maxime RT PreMix (cat. no. 25081/96, Intron Biotechnology, Seoul, Korea) containing oligo-dT primers, according to the manufacturer’s instructions. PCR amplification was performed using the synthesized cDNA as a template with a 20 μL volume consisting of DNA polymerase (cat. no. 501-025, General, Seoul, Korea) at 95 °C for 3 min for initial denaturation, followed by 35 cycles at 95 °C for 30 s, 55 °C for 30 s, and 72 °C for 30 s, and a final extension at 72 °C for 5 min. In the experiment, the reference gene used was the β-actin gene.

### 2.14. Cell Line and Cell Culture

The American Type Culture Collection served as the source for the renal tubular epithelial cell line NRK-52E. NRK-52E cells were cultured in low glucose (5.6 mM) in Dulbecco’s modified Eagle’s medium supplemented with 5% fetal bovine serum (FBS) and 1% arachidonic acid (AA) at 37 °C and 5% CO_2_. The cells were then divided into three groups: the standard glucose group (NG, 5.6 mM glucose), high-glucose group (HG, 30 mM glucose), and high-glucose (HG, 30 mM glucose) with Tenovin-1 (50 µM) group. All cell experiments were performed according to the above-mentioned protocols.

### 2.15. DAPI Nuclear Staining

The nuclear morphology and appearance of the apoptotic nucleus were confirmed by staining with 4′,6-diamidino-2-phenylindole (DAPI). NRK-52E cells were seeded and treated with HG and Tenovin-1, as stated above (Section 2.14). The cells were then fixed with paraformaldehyde for 15 min. The cells were treated with 0.1 μg/mL DAPI in phosphate-buffered saline (PBS) for 3–5 min followed by washing with Dulbecco’s PBS (DPBS). The cells were then rewashed, and nuclear morphology and apoptotic bodies were observed using fluorescence microscopy.

### 2.16. Annexin V-FITC Binding Assay

Apoptosis studies were performed using the Annexin V-FITC staining kit I (BD Biosciences, San Diego, CA, USA). The NRK-52E cells were cultured and treated as described above (Section 2.14). Subsequently, whole cells were harvested, and experimental cells were stained with annexin V-FITC (2 μL (1 mg/mL)) and propidium iodide (4 μL (1 mg/mL)) diluted with 100 μL incubation buffer. The cells were then incubated in the dark for 30 min at room temperature. Then, without washing the cells, they were analyzed using flow cytometry (Guava EasyCyte flow cytometer; Millipore, Massachusetts, USA).

### 2.17. Cell Cycle Analysis

The NRK-52E cells were cultured and treated as described in Section 2.14. The adhered cells were removed and preserved with 70% cold ethanol at 4 °C overnight. The cells were washed with cold DPBS, suspended in propidium iodide (PI) and RNAase staining solution, and incubated in the dark for 30 min at 37 °C. Finally, the cells were analyzed using flow cytometry (Guava^®^ EasyCyte flow cytometer).

### 2.18. Statistical Analysis

The results are expressed as the mean ± SD. One-way analysis of variance (ANOVA) was used to compare the means, followed by Bonferroni’s multiple comparison test, using GraphPad Prism 5 v5.0. (GraphPad Software Inc., San Diego, CA, USA). Post hoc testing was performed for intergroup comparisons using the least significant difference test. Statistical significance is indicated by ** *p* < 0.01 and * *p* < 0.05.

## 3. Results

### 3.1. Effect of Tenovin-1 on Blood Glucose and Body Weight

**Figure 2:**
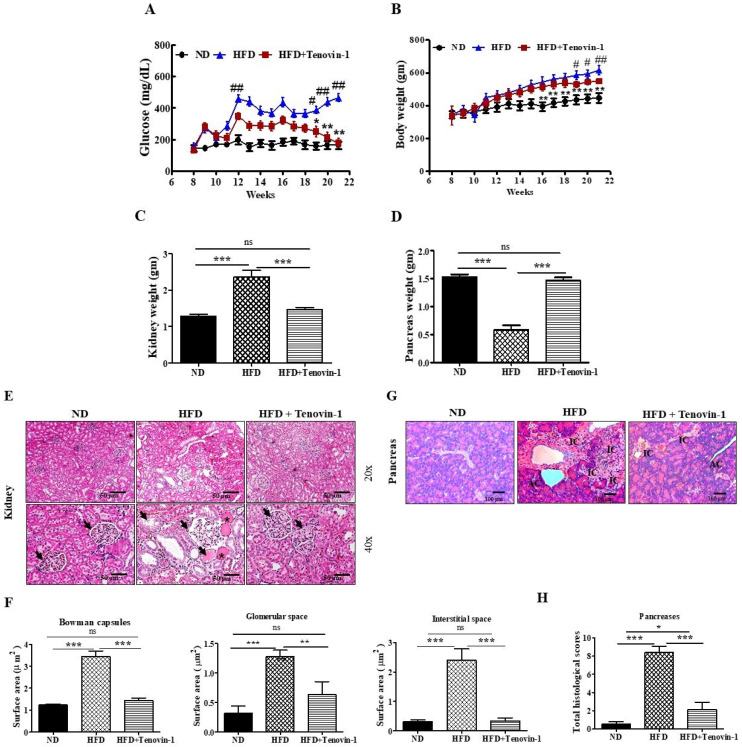
Effect of Tenovin-1 on glycemic status and microscopic appearances of the kidneys in HFD-induced diabetic rats. HFD-induced body weight gains in ZDF rats. (**A**) Effect of Tenovin-1 on fasting blood glucose concentration (mg/dL). (**B**) Body weight significantly increased in the HFD-fed rats, which was normalized following treatment with Tenovin-1. The values are presented as the mean ± S.D. of six rats per group. Statistical analysis was performed through one-way analysis of variance (ANOVA) followed by Tukey’s HSD post hoc test for multiple comparisons. * p < 0.05, ** p < 0.01, and *** p < 0.001 as compared with the group that received a normal diet (ND); *^#^ p* < 0.05 and *^##^ p* < 0.01 as compared with the HFD-fed group. ND, normal diet; HFD, high-fat diet. (**C**) Effect of Tenovin-1 on relative kidney weight. (**D**) Effect of Tenovin-1 on relative pancreas weight. (**E**) Histological alterations in kidney tissue stained with H&E stain. At 21 weeks, the expanded cortex, glomerular sclerosis (arrow heads), expansion (asterisk), and dilation were visible in the rats with HFD-induced diabetes. Tubular dilation, fibroplasia, and interstitial nodular sclerosis were observed in the medulla. HFD-fed rats treated with Tenovin-1 showed renal cortexes (RCs) of a normal size, low incidences of medullar and tubular injury, and normal histological architecture comprising thin tubules and collecting ducts (magnification 200×; bar = 50 μm). (**F**) Quantitative analysis of the Bowman capsules, glomerular space, and interstitial regions of the H&E-stained kidney sections from the experimental groups. (**G**) Representative microscopic images of the H&E-stained pancreas sections from the experimental group. The normal-diet-fed group displayed normal pancreatic architecture. In the pancreas, acinar cells and the exocrine component were tightly packed. Pancreatic lobules were divided by intact interlobular and intralobular connective tissue septa. The islet cells scattered between the acinar cells were observed to be stained less in contrast to the surrounding acinar cells. Both the endocrine and exocrine systems of the diabetic rats fed an HFD showed severe abnormalities. Small vacuoles were seen in nearly every acinar cell, and the cells were observed to be enlarged in the rats receiving an HFD; the β-cells of islets were nearly completely lost. However, the treatment of the HFD-fed rats with Tenovin-1 repaired all types of alterations. The images indicate the outcomes obtained from three animals per experimental group. (**H**) Total histological scores of the H&E-stained pancreas sections from the experimental groups (magnification 200×; bar = 50 μm). The data are the mean ± SD of duplicate experiments (6 animals/group). Statistical analysis was performed using one-way ANOVA followed by Tukey’s HSD post hoc test for multiple comparisons (*** *p* < 0.001, ** *p* < 0.01, and * *p* < 0.05). “ns” (two groups are non-significant with each other). ND, normal diet; HFD, high-fat diet.

The serum glucose levels and body weights of rats involved in this study were determined before and after the treatment with Tenovin-1. After the continuous administration of HFD, fasting serum glucose levels increased significantly in the HFD-fed rats when compared with the glucose levels in rats fed a normal cholesterol-free diet. Figure 2A shows a dramatic decrease in the fasting blood glucose levels of the HFD-fed group following Tenovin-1 treatment after 21 weeks. The above results revealed that Tenovin-1 (SIRT-1 and SIRT-2 inhibitor) has a high potential to reduce the hyperglycemic condition in HFD-induced diabetic rats. During the study, high-fat-diet-induced diabetic rats observed significant weight gain compared with the rats fed a normal diet. At the end of this experiment, the body weights of rats administered with Tenovin-1 were significantly decreased, in contrast with the rats fed a high-fat diet (Figure 2B).

### 3.2. Effect of Tenovin-1 on the Weight of Major Organs and Histological Results in ZDF Rats Fed an HFD

The major organs (kidneys and pancreas) were collected after the animals were sacrificed. The weight of the kidneys and pancreases of HFD-fed rats were significantly different, based on relative organ weight, when compared with those of rats fed a normal diet. As shown in Figure 2C, the average weight of the kidneys in the HFD-fed diabetic group was substantially greater than in the group that received a normal diet. However, the average weight of the kidneys in the group treated with Tenovin-1 was markedly lower than that in the HFD-fed diabetic group. In contrast, the average weight of the pancreas was much lower in diabetic rats fed the HFD compared with diabetic rats fed a normal diet, but there was a rise in the weight after treatment with Tenovin-1 (Figure 2D). Photomicrographs of kidney tissues from different groups are shown in Figure 2 for rats fed a normal diet. H&E staining of the kidney tissues revealed the enlargement of the glomerular tissue in HFD-fed rats, in contrast to that in rats fed a normal diet. Proximal and distal tubes were enlarged and vacuolated in HFD-induced diabetic rats compared with those in rats fed a normal diet. Additionally, the severe degeneration of vacuoles was detected in the glomerular spaces and tubules of kidneys of HFD-fed rats. The key characteristics associated with diabetic kidney damage, including glomerular basement membrane (GBM) thickening, the accumulation of lipid droplets, and podocyte fusion, were significantly improved following treatment with Tenovin-1. The general morphology of the kidney was improved, and the expanded atypical architecture of the proximal and distal tubes was restored (Figure 2E, F). Diabetic rats fed an HFD rarely showed islets with a large vacuolar area in the pancreas. However, pancreatic islets in the control group had round and clear boundaries. Few irregularly shaped islets were observed in the Tenovin-1-treated group. The results indicated that Tenovin-1 could reduce the severity of renal injury to the pancreas caused by HFD. The Tenovin-1-treated group depicted a better outcome than HFD-fed rats in terms of recovery from pancreatic impairment (Figure 2G, H).

### 3.3. Effect of Tenovin-1 on AGEs and Serum Biochemical Parameters

**Figure 3:**
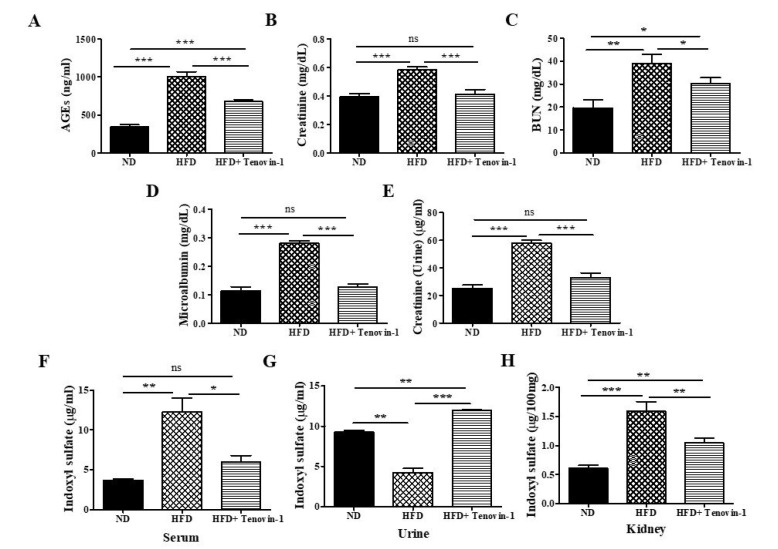
Effect of Tenovin-1 on the serum and urinary biochemical parameters of rats with HFD-induced diabetes. (**A**) Advanced glycation end products in serum (AGEs), (**B**) creatinine (sCr), and (**C**) blood urea nitrogen (BUN) levels in rats with diabetes induced by the HFD. Tenovin-1 significantly decreased the serum-level of AGEs, sCr, and BUN in the HFD-induced diabetic rats. (**D**) Changes in urinary excretions of microalbumin and (**E**) creatinine. In the HFD-induced diabetic rats, Tenovin-1 dramatically reduced the levels of the urine biomarkers microalbumin and creatinine. The concentration of 3-indoxyl sulfate (3-IS) in the HFD-fed rats was determined following Tenovin-1 administration. The 3-IS concentrations in the serum (**F**), urine (**G**), and kidney (**H**) tissues were restored significantly following treatment with Tenovin-1. The data are the mean ± SD of duplicate experiments (6 animals/group). Statistical analysis was performed using one-way ANOVA followed by Tukey’s HSD post hoc test for multiple comparisons (*** *p* < 0.001, ** *p* < 0.01, and * *p* < 0.05). “ns” (two groups are non-significant with each other). ND, normal diet; HFD, high-fat diet.

AGE accumulation in the serum was significantly higher in rats fed an HFD than in rats fed a normal cholesterol-free diet. However, the AGE content in the serum decreased following treatment with Tenovin-1 (Figure 3A). Additionally, BUN and creatinine levels were significantly higher in rats fed an HFD than in rats fed a normal diet. However, BUN and creatinine levels declined sharply following treatment with Tenovin-1 (Figure 3B,C).

### 3.4. Effect of Tenovin-1 on Urinary Biochemical Parameters

Microalbumin excretion via urine was significantly higher in HFD-fed rats compared with the rats fed a normal diet. Microalbumin concentrations decreased significantly following Tenovin-1 administration (Figure 3D). Creatinine clearance is one of the key indicators of a kidney disorder. The urinary excretion of creatinine in rats fed an HFD was dramatically higher than that in rats fed a normal diet. After Tenovin-1 treatment, creatinine levels decreased significantly (Figure 3E). A metabolite of dietary tryptophan, 3-IS, plays a notable role in nephrotoxicity. The concentration of 3-IS in the urine of HFD-fed rats was significantly lower than that in normal-diet-fed rats. However, in rats fed an HFD, the levels of 3-IS in the serum and renal tissues increased considerably. All abnormalities in the 3-IS concentrations in the serum, urine, and kidney tissues were significantly ameliorated after treatment with Tenovin-1 (Figure 3F–H).

### 3.5. Effect of Tenovin-1 on Oxidative Biomarkers of Diabetic Rats

**Figure 4:**
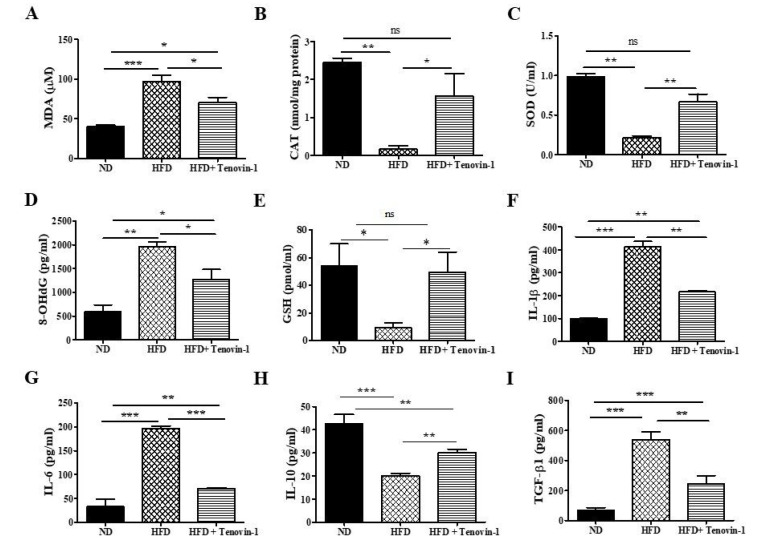
Effect of Tenovin-1 on the antioxidant enzyme activity, proinflammatory cytokines, and an anti-inflammatory cytokine in HFD-induced diabetic rats. Changes in oxidative biomarkers, such as in (**A**) levels of lipid peroxidation biomarker (malondialdehyde (MDA)) (**B**) and activities of catalase (CAT) and (**C**) superoxide dismutase (SOD), were assessed in the kidneys of HFD-induced rats. The level of an oxidative DNA damage marker, (**D**) 8-hydroxy-2′-deoxyguanosine (8-OhDG), was measured in the urine of the HFD-induced rats. The levels of measured oxidative biomarkers were restored after Tenovin-1 treatment. (**E**) Endogenous glutathione (GSH) was measured in the kidneys of HFD-induced rats. (**F**–**I**) The levels of proinflammatory cytokines (IL-1β, IL-6, and TGF-β1) were significantly decreased, and the level of anti-inflammatory cytokine (IL-10) was significantly augmented after treatment of HFD-fed rats with Tenovin-1. The data are the mean ± SD of duplicate experiments (6 animals/group). Statistical analysis was performed using one-way ANOVA followed by Tukey’s HSD post hoc test for multiple comparisons (*** *p* < 0.001, ** *p* < 0.01, and * *p* < 0.05). “ns” (two groups are non-significant with each other). ND, normal diet; HFD, high-fat diet.

Although the MDA concentration was significantly augmented in the kidney tissues of HFD-fed rats as compared with those in normal-diet-fed rats, the CAT and SOD activity was significantly decreased. After Tenovin-1 administration, the MDA content in the renal cortex was reduced, whereas the activity of CAT and SOD increased significantly in the HFD-fed rats (Figure 4A–C). The urinary concentration of 8-OHdG, a biomarker of oxidative DNA damage, increased significantly in HFD-fed rats; however, after treatment with Tenovin-1, 8-OHDG levels in the urine of HFD-fed rats showed a significant decrease (Figure 4D). The level of the GSH was markedly reduced in the kidneys of the HFD-fed rats in comparison to the normal-diet-fed rats. However, the administration of Tenovin-1 significantly restored the concentration of GSH (Figure 4E).

### 3.6. Effect of Tenovin-1 on Inflammatory Cytokines of Diabetic Rats

The levels of the pro-inflammatory cytokines IL-1β, IL-6, and TGF-β in HFD-induced diabetic rats were augmented by 3- to 4-fold, whereas the response against inflammation, based on IL-10, was inhibited. However, treatment with Tenovin-1 had inhibitory effects on all pro-inflammatory cytokines (IL-1β, IL-6, and TGF-β), whereas an opposite effect was observed for cytokine IL-10; Tenovin-1 treatment showed significantly higher levels of IL-10 in the rats fed a high-fat diet (Figure 4F–I).

### 3.7. Effect of Tenovin-1 on Urinary Biomarkers in HFD-Induced ZDF Rats

**Figure 5:**
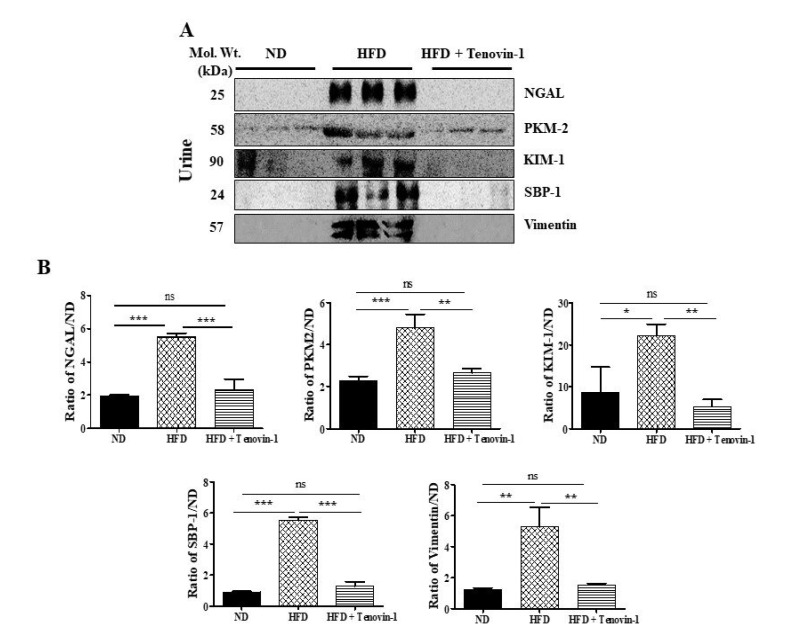
Effect of Tenovin-1 on the urinary excretion of indicators of kidney injury in rats with diabetes caused by the HFD. (**A**) Representative Western blots of NGAL, PKM-2, KIM-1, SBP-1, and vimentin expression in urine. Changes in the levels of kidney damage indicators in the rats with HFD-induced diabetes following Tenovin-1 treatment were observed. (**B**) Using Image J software, the band intensities were densitometrically quantified. The data are the mean ± SD of duplicate experiments (6 animals/group). Statistical analysis was performed using one-way ANOVA followed by Tukey’s HSD post hoc test for multiple comparisons (*** *p* < 0.001, ** *p* < 0.01, and * *p* < 0.05). “ns” (two groups are non-significant with each other). ND, normal diet; HFD, high-fat diet.

The effect of blood sugar levels on the excretion of protein-based indicators in the urine (NGAL, KIM-1, PKM-2, SBP-1, and vimentin) in diabetic rats was also investigated. Immunoblot analysis data revealed that the rats fed the HFD had much higher levels of KIM-1, NGAL, PKM-2, SBP-1, and vimentin in their urine than rats fed a normal, cholesterol-free diet; however, the expression decreased significantly after Tenovin-1 administration (Figure 5A,B).

### 3.8. Effect of Tenovin-1 on Renal Fibrosis in HFD-Induced ZDF Rats and NRK-52E Cell Line

**Figure 6:**
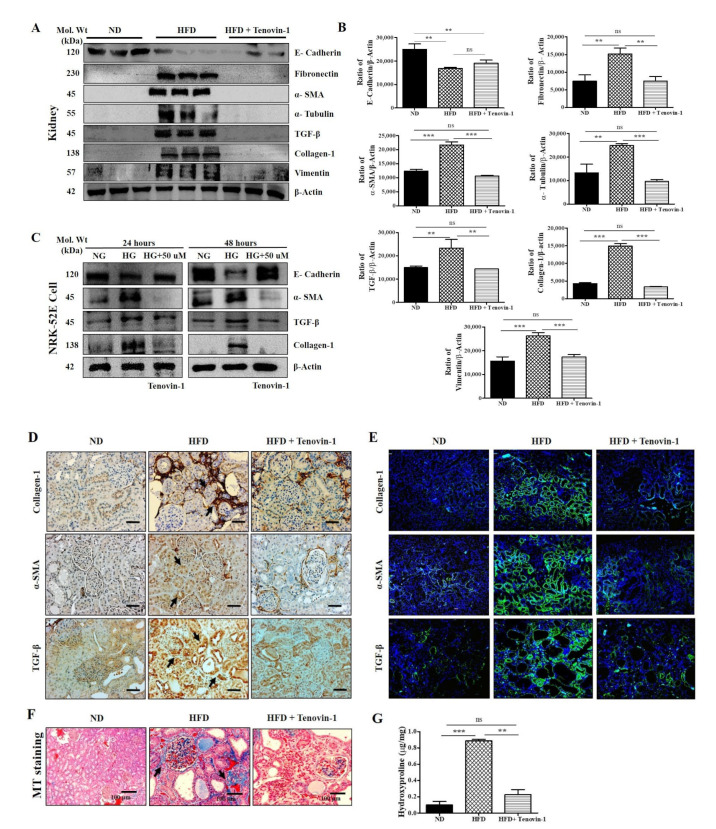
Effect of Tenovin-1 on renal fibrosis biomarkers in rats with HFD-induced diabetes. (**A**) The levels of expression of E-cadherin, fibronectin, α-SMA, α-tubulin, TGF-β, collagen-1, and vimentin were measured using Western blot analysis in experimental rat models of HFD-induced diabetes. The graph for Western blot analysis represents the results obtained from three separate experimental outcomes. β-actin was used as the loading control. (**B**) The band intensities were quantified densitometrically using Image J software. (**C**) Western blot analysis of renal fibrosis biomarkers using the NRK-52E cell lysate. (**D**) Representative immunohistochemical analysis of collagen-1, α-SMA, and TGF-β1 in HFD-induced diabetic rat kidneys. Black arrows indicate the collagen-1, α-SMA, and TGF-β1 expression levels. (**E**) Demonstrative immunofluorescence analysis of collagen-1, α-SMA and TGF-β1 in kidney tissues of HFD-induced diabetic rats. (**F**) Representative Masson’s trichrome staining of kidney sections. Black arrows represent collagen accumulation (blue color). (**G**) 4-hydroxyproline concentrations were estimated in the kidneys of rats with HFD-induced diabetes. The data are the mean ± SD of duplicate experiments (6 animals/group). Statistical analysis was performed using one-way ANOVA followed by Tukey’s HSD post hoc test for multiple comparisons (*** *p* < 0.001, ** *p* < 0.01). “ns” (two groups are non-significant with each other). ND, normal diet; HFD, high-fat diet.

Experimental and clinical studies have revealed that kidney fibrosis and inflammation are always highly correlated with HFD [18,19]. Collagen and fibronectin are crucial factors in sclerosis, followed by inflammation in kidney tissues. Therefore, we focused on the trends in molecular biomarkers associated with renal fibrosis. The levels of proteins, including α-SMA, collagen-1, α-tubulin, TGF-β, vimentin, fibronectin, and E-cadherin, were determined using Western blotting (Figure 6A, B). The results revealed that the HFD-fed diabetic rats exhibited significantly higher levels of expression of collagen-1, α-SMA, α-tubulin, TGF-β, vimentin, and fibronectin in the renal cortex than in rats fed a normal diet. However, following treatment with Tenovin-1, the level of expression of ECM proteins was significantly restored. In contrast, E-cadherin expression showed an opposite trend in the renal cortex.

Additionally, we measured the level of expression of E-cadherin, α-SMA, TGF-β, and collagen-1 proteins in the NRK-52E cell lysate using Western blot analysis. NRK-52E cells were induced with HG, and α-SMA, TGF-β, and collagen-1 expression levels were increased compared with those in NG-treated cells. However, following treatment with Tenovin-1, the protein expression levels were decreased significantly, whereas E-cadherin displayed an opposite trend (Figure 6C). The quantification of these Western blots is presented in Supplementary Figure S1. Similarly, immunohistochemical and immunofluorescence staining showed reductions in the numbers of collagen-1, α-SMA, and TGF-β-positive cells in the fibrous septa of kidneys following treatment with Tenovin-1 in HFD-fed rats. ECM accumulation is influenced by TGF-β hypertrophy, which reduces the glomerular filtration rate (GFR) and leads to CKD [20,21,22,23]. When compared with rats fed a regular normal diet, the kidneys of HFD-fed rats displayed a noticeably higher level of TGF- β expression. However, TGF-β expression was reduced following Tenovin-1 administration in HFD-fed rats (Figure 6D, E). Therefore, Tenovin-1 notably inhibited ECM production or accumulation associated with an HFD in kidneys, which inhibited the development of renal fibrosis. MT staining was performed to identify collagen accumulation in rat kidneys. Higher levels of collagen were observed in the HFD-induced diabetic rat kidneys than in the kidneys of the rats fed a normal diet, which was associated with the formation of nodules. However, the higher levels of production, as well as accumulations of collagen, were significantly restored following Tenovin-1 administration to HFD-induced diabetic rats (Figure 6F). To confirm the role of fibrosis markers in renal fibrosis, a 4-hydroxyproline assay was performed. The 4-hydroxyproline assay was used to determine the production and accumulation of ECM, as well as fibrosis severity. The alteration of ECM proteins was significantly restored in HFD-induced diabetic kidneys following treatment with Tenovin-1. The 4-hydroxyproline assay was performed to provide supporting evidence (Figure 6G).

### 3.9. Effects of Tenovin-1 on Renal Apoptosis in Diabetic Rats and HG-Induced NRK-52E Cell Line

**Figure 7:**
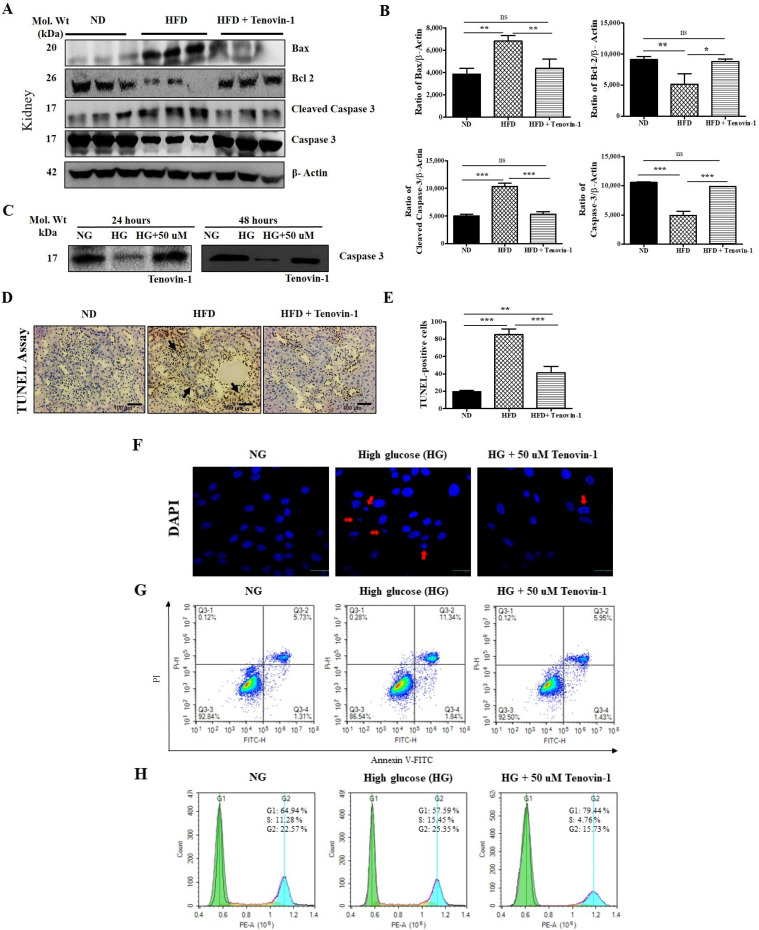
Effects of Tenovin-1 on the expression of proteins linked to apoptosis in the kidney tissue of diabetic rats fed a high-fat diet. (**A**) The levels of expression of Bax, Bcl2_,_ cleaved caspase-3, and caspase-3 were analyzed via Western blot analysis using experimental rat models of HFD-induced diabetes. β-actin was used as the loading control. (**B**) Using Image J software, the band intensities were densitometrically quantified. (**C**) Western blot analysis of caspase-3 for NRK-52E cells. (**D**) Apoptosis was analyzed by TUNEL staining of the kidney tissues from HFD-fed rats. Original magnification: 200×; scale bar: 100 μm. (**E**) Determined TUNEL-positive score index. (**F**) Representative 4′-6-diamidino-2-phenylindole (DAPI) staining to assess apoptotic cell death. (**G**) Analysis of apoptotic (Annexin V/PI) assay to determine the proportions of late-stage apoptotic cells. (**H**) Flow cytometry analysis for cell cycle profiles in high-glucose-induced NRK-52E cell lines. The data are expressed as the mean ± SD of duplicate experiments (6 animals/group). Statistical analysis was performed using one-way ANOVA followed by Tukey’s HSD post hoc test for multiple comparisons (*** *p* < 0.001, ** *p* < 0.01, and * *p* < 0.05). “ns” (two groups are non-significant with each other). ND, normal diet; HFD, high-fat diet.

To detect the level of expression of apoptotic proteins, we performed Western blot analyses using kidney tissues from HFD-induced ZDF rats. In HFD-induced rats, Bax and cleaved caspase-3 expression levels were significantly upregulated, which then decreased after the administration of Tenovin-1. Conversely, the levels of expression of Bcl2 and caspase-3 were decreased in HFD-fed rats, followed by increases after treatment with Tenovin-1 (Figure 7A,B). The lower levels of caspase-3 expression in the presence of high glucose were downregulated significantly followed by treatment with Tenovin-1 for NRK-52E cells (Figure 7C). Chromatin condensation was examined using the TUNEL assay, and the results were consistent with the severity of kidney damage, as displayed in Figure 7D. Kidney apoptosis was observed in rats fed an HFD. Apoptotic cell death significantly decreased following treatment with Tenovin-1 in HFD-fed diabetic rats (Figure 7D,E).

Additionally, DAPI staining was performed to assess HG-induced apoptotic cell death in NRK-52E cells. Apoptotic cells were compared between NG- and HG-induced NRK-52E cells. HG-treated cells showed fragmented or condensed apoptotic nuclei. After treatment with Tenovin-1, condensed chromatin and apoptotic nuclei were resolved (Figure 7F). Subsequently, we performed an Annexin V/PI assay to determine the proportions of late-stage apoptotic cells. We compared the proportions of late apoptotic cells in NG- and HG-induced groups; due to the high glucose levels, the number of apoptotic cells in the HG-induced group was increased in comparison with that in the NG-induced group. However, after treatment with 50 µM Tenovin-1, the number of apoptotic cells decreased (Figure 7G). Flow cytometry was performed to determine the cell cycle profiles in the HG-induced NRK-52E cell lines. After HG induction, the cell population in the G1 phase decreased as compared with that in the S-phase in NRK-52E cells compared with in NG. In contrast, the proportion of G1-phase cells increased, and that of S-phase cells decreased in the Tenovin-1-treated group (Figure 7H).

### 3.10. Effect of Tenovin-1 on Sirtuins and Claudin-1 in Renal Cortexes of HFD-Fed Rats

**Figure 8:**
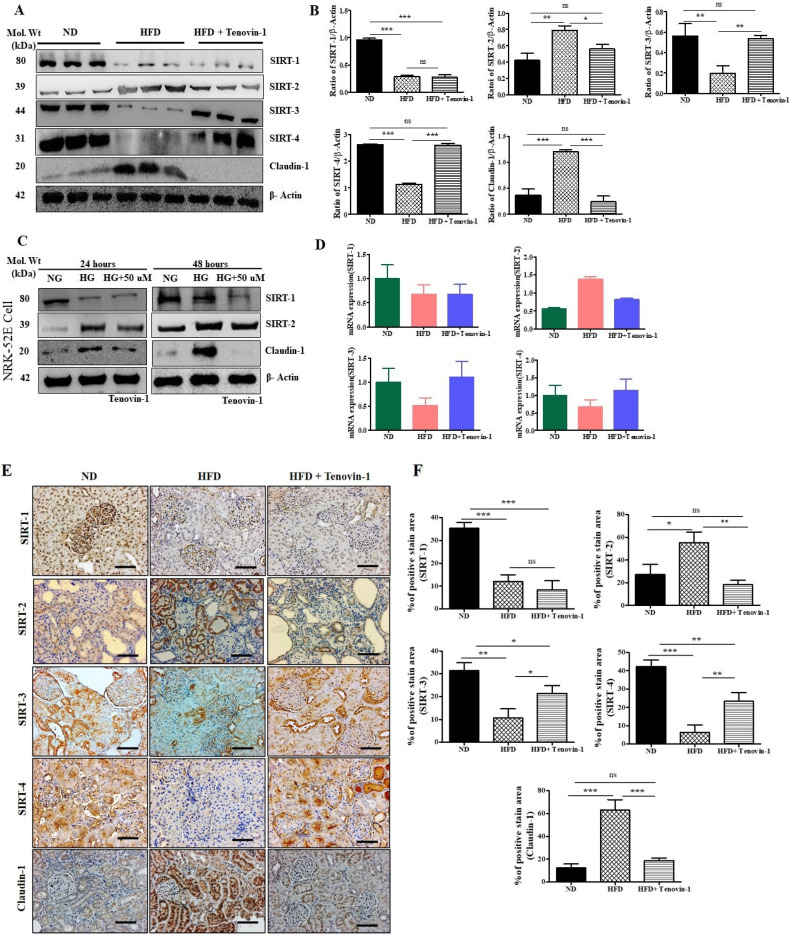
The effects of Tenovin-1 on SIRT and claudin-1 expression were measured by Western blot analysis using experimental rat models of HFD-induced diabetes. (**A**) The levels of SIRT1, SIRT2, SIRT3, SIRT4, and claudin-1 were analyzed using Western blotting in the kidneys of rats with HFD-induced diabetes. β-actin was used as the loading control. The outcomes of three different experiments were combined in the Western blotting results. (**B**) The band intensities were measured densitometrically. (**C**) Western blot analysis of SIRT and claudin-1 levels was carried out using the NRK-52E cell lysate. (**D**) mRNA expression levels of SIRTs (SIRT1, SIRT2, SIRT3, and SIRT4) were investigated using qPCR. (**E**) Representative immunohistochemical analyses of SIRT1, SIRT2, SIRT3, SIRT4, and claudin-1 in the kidneys of HFD-induced diabetes rats. Original magnification: 200×; scale bar: 50 μm. (**F**) Percentage (%) of positive staining area of SIRTs (SIRT1, SIRT2, SIRT3, and SIRT4). The data are the mean ± SD of duplicate experiments (6 animals/group). Statistical analysis was performed using one-way ANOVA followed by Tukey’s HSD post hoc test for multiple comparisons (*** *p* < 0.001, ** *p* < 0.01, and * *p* < 0.05). “ns” (two groups are non-significant with each other). ND, normal diet; HFD, high-fat diet.

Considering the role of SIRT proteins in the progression of kidney fibrosis and in HFD-fed rats, we also explored the levels of expression of SIRT1, SIRT2, SIRT3, SIRT4, and claudin-1 in DN rats. To determine whether the rise in SIRT expression was related to renal disease, the levels of SIRT1, SIRT2, SIRT3, and SIRT4 were assessed using Western blotting. According to the results, the levels of SIRT1, SIRT3, and SIRT4 in the renal cortexes of HFD-fed rats were significantly decreased, whereas SIRT2 and claudin-1 levels were upregulated, in comparison with those in normal rats. However, treatment with Tenovin-1 significantly downregulated SIRT1, SIRT2, and claudin-1 levels (Figure 8A). The intensities of the bands obtained through Western blot analysis were measured and are shown as bar graphs (Figure 8B). SIRT1 and SIRT2 levels in the NRK-52E cell line were evaluated using Western blot analysis. In HG-induced NRK-52E cells, SIRT-1 levels were decreased and SIRT2 was increased compared with those in the NG-induced NRK-52E cells. However, treatment with Tenovin-1 gradually decreased the levels of SIRT-1 and SIRT-2, as indicated by the Western blot analysis results (Figure 8C). The quantification of these Western blots is presented in Supplementary Figure S2. Additionally, the mRNA levels of SIRT1, SIRT3, and SIRT4 were reduced, whereas the SIRT2 level was increased in the kidneys of HFD-fed diabetic rats. However, the levels of expression of SIRT1 and SIRT2 decreased, whereas SIRT3 and SIRT4 increased following treatment with Tenovin-1 in HFD-fed rats (Figure 8D). To confirm the protective effects of Tenovin-1 on renal fibrosis in HFD-induced diabetic rats, immunohistochemical analysis was performed to detect the levels of expression of SIRT1, SIRT2, SIRT3, SIRT4, and claudin-1 in kidney tissues. SIRT1, SIRT2, SIRT3, SIRT4, and claudin-1 were expressed in the glomeruli and interstitial areas of the kidneys of normal-diet-fed rats. However, the highest level of expression was observed for SIRT2 and claudin-1, whereas the levels of SIRT1, SIRT3, and SIRT4 were observed to be decreased in the kidney tissues of HFD-fed rats compared with those of rats fed a normal diet. However, expressions were restored following Tenovin-1 treatment, thus corroborating the immunoblot data. The levels of expression of SIRT1, SIRT2, and claudin-1 were downregulated, whereas those of SIRT3 and SIRT4 were upregulated, following treatment with Tenovin-1, corroborating the data obtained using immunoblotting (Figure 8E,F).

### 3.11. Effect of Tenovin-1 on the Phosphorylation Levels of EGFR, PDGFRβ, and STAT3 in Kidneys of HFD-Induced ZDF Rats

**Figure 9:**
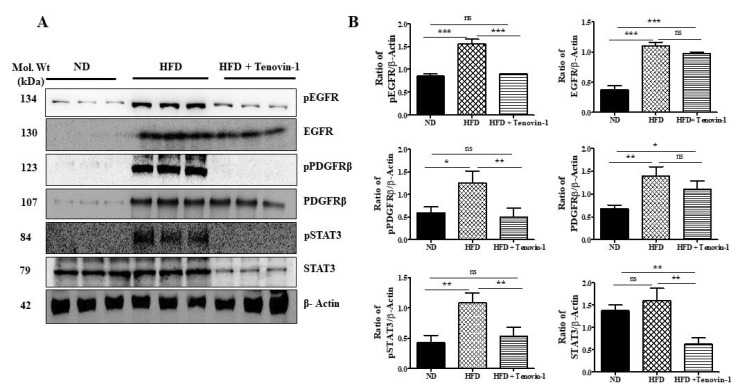
Effect of Tenovin-1 on EGFR, PDGFRβ, and STAT3 phosphorylation in the kidney tissues of HFD-fed diabetic rats. (**A**) The total and phosphorylated levels of EGFR, PDGFRβ, and STAT3 were examined using Western blot analysis. β-actin was used as the loading control. Three separate Western blot experiments were used to obtain the results. (**B**) The intensities of the bands were analyzed densitometrically using Image J software. The data are the means ± SD of duplicate experiments (6 animals/group). Statistical analysis was performed using one-way ANOVA followed by Tukey’s HSD post hoc test for multiple comparisons (*** *p* < 0.001, ** *p* < 0.01, and * *p* < 0.05). “ns” (two groups are non-significant with each other). ND, normal diet; HFD, high-fat diet.

Using Western blot analysis, the effects of Tenovin-1 on EGFR and PDGFR activation in the renal cortexes of HFD-induced diabetic rats and the phosphorylation levels of EGFR and PDGFR were assessed. According to the results, EGFR and PDGFRβ phosphorylation was increased in the kidneys of HFD-fed diabetic rats; however, Tenovin-1 administration markedly inhibited the phosphorylation without altering their expression. EGFR and PDGFRβ levels were increased in HFD-fed rats. Furthermore, rats fed an HFD had higher levels of EGFR and PDGFRβ in their renal cortexes (Figure 9A,B). The data are consistent with the findings of published reports [14,15], stating that one of the factors regulating the expression of these tyrosine kinase receptors is SIRT1 inhibition. The effect of Tenovin-1 on the phosphorylation of STAT3 in the kidneys of HFD-fed rats was investigated. Our results revealed that STAT3 and p-STAT3 expression was markedly upregulated in the kidneys of HFD-induced diabetic rats. However, the phosphorylation of STAT3 was dramatically reduced after Tenovin-1 administration (Figure 9A,B).

## 4. Discussion

DN is a major feature of end-stage renal disease (ESRD). By 2025, approximately 300 million people will experience DM-related complications. DN occurs in approximately 30–40% patients with type 2 diabetes and 15–25% patients with type 1 diabetes [24]. Consequently, effective treatments of DN are in high demand. However, there is scant published content that exhibits the beneficial role of SIRT inhibitors in DN and attempts to establish a mechanistic approach. SIRT antagonistic therapy has more satisfactory effects on diabetic-induced renal injury than conventional medicine [15].

Zucker diabetic fatty (ZDF) rats are a widely used model to study diabetes and obesity [25]. Male ZDF rats exhibit functional features of T2DM including hyperphagia, severe hyperglycemia, hyperlipidemia, hyperinsulinemia, poor glucose tolerance, hypertension, and heart failure [25,26]. ZDF rats have hyperglycemia and dyslipidemia from an early age (2 months), which worsens as they age (8 month of age). ZDF rats’ renal histology at 8 months revealed pathological alterations, including glomerular sclerosis with thickening of the Bowman capsule and retraction of the tuft, tubular atrophy and dilatation, and hyaline casts [27,28]. The ZDF rats were fed a high-fat diet in order to make the nephropathy more severe and shorten the study’s duration. As a result, modifications may be necessary in order to use the model as effectively as possible in biomedical or preclinical research. Some studies revealed that male fa/fa rats in the ZFD mellitus strain are fertile; they exhibit the development of hyperglycemia, obesity, and hyperlipidemia at an age of as young as 10 weeks; and they attain 100% DN at approximately 20 weeks of age. However, the development of nephropathy in ZDF rats within 12 weeks in the present study was faster than that in most other models of diabetes with chronic renal insufficiency [16,18,29,30,31].

The present study demonstrated that Tenovin-1 exerts protective activity against DN in HFD-induced ZDF rats. Histological alterations of the renal cortex are associated with fibrosis in kidney and urinary 8-OHdG excretion, which have a direct relationship with higher glucose levels in HFD-fed rats [16,32,33,34]. To the best of our knowledge, this is the first study which demonstrates that Tenovin-1 prevents the development of HFD-induced renal fibrosis. One published report has revealed that the levels of expression of SIRT1/2 are altered under different physiological conditions [14]. Generally, SIRT1 expression is reduced under the conditions of metabolic disorders, such as diabetes and cardiovascular disease.

The function of SIRTs, specifically SIRT1, on tissue-specific fibrosis is a topic of controversy. In this study, we also evaluate that an inhibitor of SIRT1 and SIRT2, Tenovin-1, might play a role in the reduction in renal fibrogenesis. SIRT1 and SIRT2 belong to the NAD+-dependent family of histone deacetylases (HDACs), i.e., Class III HDACs. HDACs play a role in the catalysis of acetyl functional group removal from lysine residues of histone and non-histone proteins [14,35,36]. Currently, the molecular understanding for class III HDAC-prompted regulations on growth factor receptors is not clearly known. However, according to our hypothesis, it is possible that the inhibition of SIRT1/2 by their inhibitors prevents their deacetylation activity, thus reducing the phosphorylation of signaling molecules and causing the dephosphorylation of epidermal growth factor receptor (EGFR), platelet-derived growth factor receptor-*β* (PDGFRβ), and the signal transducer and activator of transcription 3 associated with fibrogenesis. Thus, the downregulation of these proteins might be associated with the HDAC activity of SIRT1 and SIRT2. The effect of SIRT1 downregulation leading to a protective effect might be due to its combined inhibition of the phosphorylation of the signaling proteins along with a positive contributor, SIRT2. However, this SIRT1/2 inhibition reducing the phosphorylation of these signaling molecules needs to be further investigated.

The effect of SIRT2 on diabetic nephropathy has been less studied clinically. In one study on the function of SIRT2 in kidney injury, it was found that SIRT2 controls the activity of the protein kinase phosphatase1, which is responsible for the acute renal injury caused by cisplatin [37]. The pharmacological suppression of SIRT2 can lead to a decrease in renal interstitial fibrosis in UUO models. SIRT2 also participates in the activation of fibroblasts and TIF, mediated by regulating the MDM2 pathway [38]. In mice deficient in SIRT2 function, improvements in renal function and tubular damage were observed in cisplatin-induced AKI after treatment with lipopolysaccharide [39].

Ponnusamy et al. (2015) showed that renal fibrosis develops under the activity of an SIRT1 activator, SRT1720. Additionally, YK-3-237, an SIRT1 activator, boosts the activation of renal interstitial fibroblasts, as indicated by the upregulation of α-SMA and fibronectin expression [36]. However, the mechanistic approaches of SIRT1 activators in fibrotic tissue disorders remain unclear and may be related to their non-specific characteristics. Therefore, the functional roles of SIRTs in the regulation of tissue-specific fibrosis remain controversial.

Generally, SIRT1 exhibits a protective effect in kidney diseases [40,41,42]. However, recent studies have revealed that the SIRT1 agonists available today may not be specific for the SIRT1 receptor [43,44]. Additionally, SIRT1 agonists have both beneficial and detrimental effects [45]. Hasegawa et al. (2013) explained that albuminuria manifested in proximal tubule (PT)-specific mice after knocking out SIRT1. To disrupt glomerular filtration, cause podocyte dysfunction, and cause albuminuria in mice, the downregulation of SIRT1 expression in PT caused the overexpression of claudin-1 in the podocytes. However, Liu et al. (2014) revealed that for SIRT1 knockout animals with a specificity for podocytes, albuminuria did not manifest in the initial state [46]. More severe kidney injury was observed in cases of diabetic mice. These differences between the two models raise the possibility that SIRT1 might support various PT and podocyte functions. To understand the cellular mechanism behind the phenotypic differences between these SIRT1 loss-of-function mice, additional research is required.

According to a previous studies, the number of acinar cells and sizes of the pancreatic islets decreased sharply in HFD-fed rats [16,47]. In this study, the islets of rats fed a high-fat diet were shrunken and damaged, whereas lymphocytic infiltration was identified in the pancreas. However, the damaged architecture of the pancreas was restored by treatment with Tenovin-1. Therefore, we suggest that Tenovin-1 could recover insulin sensitivity and prevent pancreatic damage without altering insulin secretion. Taken together, we found that high glucose, triglyceride, and cholesterol levels in the blood were observed in the HFD-fed rats. Therefore, due to the necrotic effects on pancreatic beta cells, which limit insulin release, HFD ZDF rats are commonly utilized as models for type II diabetes in humans [16,48,49,50]. These observations also revealed abnormalities in renal function and glomerular injury in HFD-induced rats [15,16,18,22]. In the current study, Tenovin-1 had a protective role in HFD-induced renal dysfunction. Microalbumin is generally considered to be a key marker of diabetic renal dysfunction and is excreted in urine [51,52]. Similarly, the urinary excretion of microalbumin was higher in HFD-fed rats, whereas Tenovin-1 dramatically reduced the urinary microalbumin levels.

Prior reports revealed that high levels of PKM2, SBP-1, NGAL, and KIM-1 were significantly excreted in the urine of rats or diabetic patients with renal dysfunction [53,54,55]. In HFD-induced ZDF rats, the urinary excretion of these protein-based biomarkers was significantly reduced by Tenovin-1 treatment. Furthermore, 3-IS is a novel biomarker of nephrotoxicity, and it is generally detected in the serum of patients with acute kidney injury (AKI) and CKD [56,57]. In addition, it is actively secreted from proximal tubules; therefore, decreased concentrations of 3-IS in urine indicate tubular damage [8,56]. Our previous study showed that the concentrations of 3-IS in urine decreased sharply in AKI [58]. In the present study, 3-IS contents in the kidney tissues and serum of HFD-fed rats were significantly high, whereas the contents in urine decreased sharply; however, the administration of Tenovin-1 restored 3-IS concentrations to nearly normal levels.

ROS production is a key factor in the development of diabetic complications, particularly DN [18,59,60]. Therefore, antioxidant therapy offers novel therapeutic approaches for patients with DN. ROS hinder the protective mechanisms of antioxidants at the cellular level. In accordance with ROS formation, CAT facilitates the neutralization of ROS via SOD [61]. Superoxide is converted by SOD to hydrogen peroxide, a less reactive ROS, which CAT then further reduces to water. MDA is a significant biomarker of the free radical scavenging potency induced by lipid peroxidation [20]. Generally, high levels of MDA are detected in the renal cortex, proximal tubules, and mesangial cells [61]. The elevated MDA levels were reduced in diabetic rats administered Tenovin-1. Therefore, Tenovin-1-treated HFD-induced diabetic rats exhibited increased activities of SOD and CAT, suggesting that Tenovin-1 has antioxidant capacity. A significant indicator for oxidative DNA damage and overall oxidative stress is 8-OHdG [33,62,63]. Higher concentrations of 8-OHdG in the urine of diabetic rats are associated with DN development [64]. According to a previous study, high 8-OHdG excretion is directly linked with microalbumin levels in DN patients [65]. In HFD-fed rats, the suppression of AGE formation is a potential therapeutic tool for the control of DN. In the present study, AGE formation was significantly inhibited through treatment with Tenovin-1. Therefore, we propose that Tenovin-1 ameliorates DN by preventing the generation and buildup of AGEs as well as oxidative damage to lipids and DNA in the renal cortex, highlighting the strong antioxidant potential of Tenovin-1. In addition to deteriorating mitochondrial structure and function, oxidative stress causes renal fibrosis and apoptosis [66].

Kidney fibrosis and tubular injury are complex pathological processes that augment oxidative stress, inflammation, apoptosis, and autophagy. The cellular mechanisms involved in renal fibrosis include the cell death process in the form of apoptosis or cellular senescence [67]. Using the TUNEL assay, we observed that Tenovin-1 suppressed apoptotic cell death. Hyperglycemia-induced cells showed fragmented or condensed apoptotic nuclei compared with culture under a normal glucose condition in the NRK-52E cells. However, HG-induced cells, after treatment with Tenovin-1, showed a reduction in apoptotic bodies. Flow cytometric analysis indicated the stimulation of apoptosis and cell cycle arrest in the NRK-52E cells. The gradation of cell damage is determined by cell cycle progression [68]. Following stimulation with HG, the proportion of cells in the G1 phase was reduced, and the cells in the S-phase were augmented significantly in NRK-52E cells compared with those of NG. In contrast, however, the percentage of cells in the G1 phase increased, whereas the percentage of cells in the S-phase decreased after Tenovin-1 treatment.

TGF-β is a crucial factor in renal fibrosis due to its production and alteration in the renal tubules [20,21,22,23]. Diabetic conditions can trigger the TGF-β pathway, which is majorly responsible for the induction of fibrosis in the kidneys [69]. ROS and TGF-β lead to glomerulosclerosis followed by hyperglycemic conditions [7]. Additionally, TGF-β1 is responsible for the proliferation and growth of basement membrane cells [70,71]. A previous report revealed that humans and animals with chronic renal disorders associated with fibrosis have highly expressed levels of TGF-β1 [70,71]. In the present study, we found that the expression of TGF-β1 was significantly reduced in HFD-induced rats following Tenovin-1 treatment. Moreover, Tenovin-1 decreased IL-6 levels in a dose-dependent manner. Increases in IL-6 are involved in the proliferation of mesangial cells, the alteration of endothelial permeability, and glomerular membrane thickening [19]. IL-1β is another important cytokine parameter in DN studies, and considerably increased IL-1β concentrations have been observed in the sera and kidneys of db/db mice [72]. In the present study, IL-1β levels were markedly increased in the HFD-induced diabetic rats, but Tenovin-1 decreased serum IL-1β levels significantly. The IL-10 level is a vital parameter in DN progression, and high levels of IL-10 protect renal dysfunction and reduce inflammation in patients with CKD [73]. In the present study, IL-10 levels were significantly decreased in HFD-fed rats, whereas the administration of Tenovin-1 considerably augmented IL-10 concentrations in HFD-fed rats.

The pathophysiology of kidney fibrosis is influenced by three major factors: ECM protein accumulation, mesangial cell matrix deposition, and GBM thickening [74]. Particularly, renal fibroblast proliferation, which results in the myofibroblast phenotype and ECM deposition, is linked to interstitial fibrosis [15]. ECM overproduction in the renal cortex is attributed to the presence of α-SMA-positive myofibroblast phenotypes in kidneys [16,75,76]. In this study, higher expression levels of α-SMA and vimentin proteins were observed in HFD-fed rats than in rats fed a normal diet. However, the levels of expression of α-SMA, collagen-1, and E-cadherin proteins in cell lysates were downregulated after treatment with Tenovin-1, which corroborates the immunoblot data for tissue analysis. Based on these results, we suggest that that Tenovin-1 had inhibitory effects on the expression of α-SMA, TGF-β, and collagen-1, which are related to the ECM in diabetic-induced renal fibrosis. Published data have revealed that a higher level of α-SMA accelerates the contractile performance of fibroblasts [77]. Moreover, the expression levels of fibronectin and collagen-1 were also reduced following Tenovin-1 treatment, which indicates the reversal of kidney fibrosis. Moreover, the atypical architecture associated with kidney injury in HFD-fed rats recovered following Tenovin-1 therapy.

The development of renal fibrosis is not only dependent on the activation of EGFR and PDGFRβ signaling pathways, but also on TGF-β pathways [71,78,79,80]. Therefore, EGFR and PDGFRβ have been assumed to be directly associated with fibroblast activation and fibrogenesis, followed by kidney injury. Furthermore, EGFR signaling is a crucial factor involved in the regulation of renal epithelial cell proliferation and dedifferentiation [71,81]. EGFR has been identified as a critical link in the regeneration and development of the kidneys [82,83,84]. Our recent study revealed that a potent SIRT1 inhibitor, EX527, blocks the phosphorylation of EGFR and PDGFR and decreases renal fibroblast activation in an HFD-induced ZDF rat model [16]. Additionally, EX527 blocks the STAT3 signaling pathway, which suppresses the expression of downstream inflammatory mediators [16]. Our study showed that Tenovin-1 can nullify the phosphorylation of EGFR, PDGFR, and STAT3. Furthermore, blocking the phosphorylation of PDGFR, EGFR, and STAT3 also inhibited the expression of fibroblast markers, such as TGF-β, α-SMA, and collagen-1. The fibroblast indicators mentioned above are important clinical characteristics that are understood to be parts of a similar ultimate pathway in all types of CKDs [85].

Due to its direct correlation with renal fibrosis, STAT3 is a critical element in inflammation. Tenovin-1 attenuated inflammation by blocking STAT3 activation and then mitigated kidney fibrosis in DN. Thus, the downregulation of these receptors may clarify the antifibrotic potentiality of Tenovin-1 in HFD-induced diabetic rats. SIRT1 and SIRT2 mediate renal interstitial fibroblast activation, proliferation, and advancement of renal fibrogenesis, indicating that SIRT1 and SIRT2 inhibitors may be helpful for the treatment of CKD. The blocking of SIRT1/2 also inhibits the phosphorylation of STAT3 in both cultured renal fibroblasts and in obstructed kidneys [8,15]. Additionally, it was shown that SIRT1/2 inhibitors reduced STAT3 phosphorylation in the sham-operated kidney in addition to inhibiting UUO-induced STAT3 phosphorylation in the kidney.

Previous studies have revealed that STAT3 phosphorylation is inhibited by blocking SIRT1/2 in cultured renal fibroblasts, obstructed kidneys, and diabetic kidneys [15,16]. The beneficial role of Tenovin-1 may be facilitated by its capacity to inactivate EGFR and block the STAT3 signaling pathway. Following renal damage, STAT3 expression is activated and increased in renal interstitial fibroblasts [86]. Phosphorylated STAT3 is highly expressed in the interstitial fibroblasts of kidneys with fibrosis [16,86]. However, it remains unclear how SIRT1/2 controls STAT3 activity and expression, even though the inactivation of SIRT1/2 can encourage or augment protein acetylation. Renal fibroblasts treated with the class I/II HDAC inhibitor also showed STAT3 acetylation. Therefore, the acetylation of STAT3 plays a critical role (positive or negative) in controlling numerous aspects of STAT signaling [87]. Inactivating the p65 subunit or lowering ROS generation through deacetylation enables SIRT1 to exert its anti-inflammatory capabilities. [88,89]. Relatively low levels of expression of SIRT1 have also been observed in renal tissues [90,91]. SIRT1 has dual cellular localization and can be found in both the nucleus and the cytoplasm [92]. SIRT1 is mainly present in the nucleus, but there are also small amounts of SIRT1 distributed in the cytoplasm [93]. In addition, research on Resveratrol, an activator of SIRT1, and its impact on diabetic nephropathy, demonstrates Sirt1 expression in adult kidneys of humans, mice, and rats, with SIRT1 distribution seen in the nucleus of the glomeruli. Sirt1-stained podocytes, endothelial cells, and mesangial cells were present in glomeruli from all three species [94]. The IHC results of SIRT1 in our investigation, which demonstrate the distribution of SIRT1 in both glomerular and non-glomerular portions of healthy and sick rat kidney tissues, are comparable with the results of this study. Other findings also demonstrated the nuclear and cytoplasmic expression of Sirt1 in both glomerulus and tubular cells [95].

The upregulation of SIRT2 was found in HFD-fed rats compared with rats fed a normal diet [96,97]. The present study revealed that SIRT1 and claudin-1 expressions showed opposite trends in the HFD-fed rat kidneys. Although SIRT1 expression was significantly decreased in the HFD-fed rats, the expression of claudin-1 was augmented. However, following Tenovin-1 treatment, the expression of SIRT1 and claudin-1 proteins was markedly decreased in rats administered the HFD. SIRT1 has been shown to ameliorate AKI and CKD [41,42]. However, the pharmacological actions of different isoforms of SIRT1 (ant-) agonists in the pathophysiology of kidney fibrosis are not clearly understood. Another recent study revealed that SIRT1 agonists are non-specific for SIRT1 receptors [43,44]. Additionally, agonists of SIRT1 have both preventive and destructive effects.

It has been revealed that the downregulation of SIRT2 suppresses fibrosis in different organs, demonstrating the protective effects [96,97].

Another Sirtuin family protein homologue, SIRT3, controls mitochondrial activity by scavenging ROS to prevent apoptosis. In diabetes-related kidney diseases, SIRT3 reduces oxidative-stress-related inflammation [98,99,100]. Reduced ROS production and attenuated inflammation creation are intimately related to SIRT3’s anti-inflammatory action. By activating SOD, calorie restriction by SIRT3 lowers oxidative damage [99,100].

SIRT4 is particularly crucial in kidney disease when it comes to mitochondrial function. Increased mitochondrial membrane potential and decreased ROS generation were seen in conjunction with the increased proliferation and suppression of apoptosis caused by SIRT4 overexpression. Under hyperglycemic circumstances, SIRT4 overexpression may prevent podocyte death and lessen podocyte damage. Additionally, SIRT4 overexpression reduced IL-1 and IL-6 production [8]. These findings suggest the involvement of SIRT4 in the inflammatory response associated with diabetic nephropathy.

By reducing mitochondrial oxidative stress and inflammatory cytokine production, Tenovin-1 may prevent renal fibrosis by upregulating SIRT3 and SIRT4, and downregulating Claudin 1. However, Tenvoin-6, a water-soluble analog of Tenovin-1 has a little or no effect as an inhibitor of SirT3 or SIRT4 in vitro, in contrast to SirT1 and SirT2, despite the high level of sequence similarity between these three class-I Sirtuins [11]. The increase in the SIRT3 and SIRT4 levels, however, might be due to a concomitant effect of SIRT1/2 inhibition on Sirt3 and SIRT4 [14,16].

Our previous study revealed that SIRT1 downregulation by EX-527 had a protective effect against hyperglycemia-induced kidney fibrosis, facilitated by proinflammatory cytokines, oxidative stress pathways, and cellular signaling pathways that are associated with renal fibrosis [16]. However, additional research is necessary to determine the underlying cellular mechanisms of the disparities in phenotype between SIRT1 loss-of-function models. In addition, the therapeutic approaches of SIRT1 and SIRT2 in HFD-induced DN have remained unclear; therefore, in the present study, we evaluated the effects of SIRT1 and SIRT2 inhibitors on renal fibrotic disorders.

## 5. Conclusions

In the present study, we exhibited the use of an in-house HFD-induced glucose metabolism disorder and renal injury rat model. To the best of our knowledge, no previous study has examined the protective effect of Tenovin-1 against hyperglycemia-induced DN. This process is mediated by oxidative stress and STAT3 cellular signaling pathways involved in renal fibrosis. Our results emphasize that Tenovin-1 might inhibit EGFR/PDGFR phosphorylation and attenuate renal fibrosis in HFD-induced rat models. Additionally, Tenovin-1 may exert its positive effects by inhibiting the activation of TGF-β and/or other growth factor receptors, which could then deactivate the STAT3 signaling pathway. The STAT3 pathway may be pharmacologically blocked to prevent the expression of downstream inflammatory mediators. Therefore, anti-fibrotic and anti-inflammatory actions may provide a protective effect against DN. Overall, our findings do not entirely refute the role of the SIRT1 and SIRT2 pathways in DN disorders, but offer insights into the pathophysiological roles of SIRT1 and SIRT2 in nephropathy. Tenovin-1 exerts potential renoprotective actions in rats with HFD-induced diabetes. Additionally, the findings of the present study could lead to the development of novel treatment strategies for DN.

## Data Availability

Not applicable.

## Supplementary Materials

The following supporting information can be downloaded at: https://www.mdpi.com/article/10.3390/antiox11091812/s1; Figure S1: Band intensities of ECM proteins from in-vitro analysis of NRK-52E cells were densitometrically quantified; Figure S2: Band intensities of SIRTs and claudin-1 from in-vitro analysis of NRK-52E cell lysates were densitometrically quantified.
