# Supplementary material for: Tenovin-1 Ameliorates Renal Fibrosis in High-Fat-Diet-Induced Diabetic Nephropathy via Antioxidant and Anti-Inflammatory Pathways
Source: Antioxidants (Basel). 2022 Sep 14;11(9):1812. doi: 10.3390/antiox11091812 (PMC9495519; doi:10.3390/antiox11091812)
Supplement: Supplementary file 1 [file antioxidants-11-01812-s001.zip › antioxidants-1834716-supplementary.pdf]

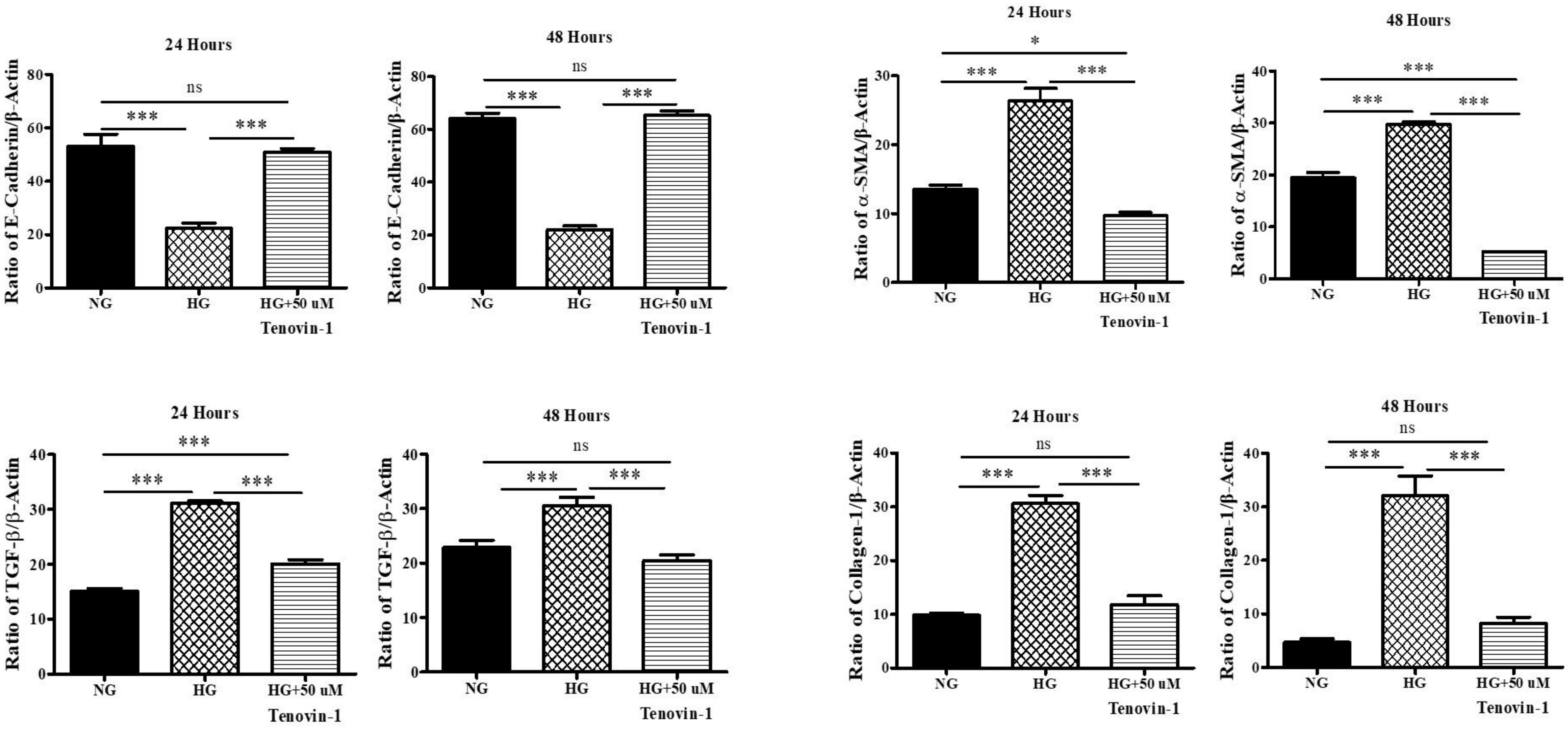

Figure S1. Band intensities of ECM proteins from in-vitro analysis of NRK-52E cells: Band intensities of ECM proteins from in-vitro analysis of NRK-52E cells were quantified by densitometric analysis and have been presented as relative ratio of three repetitive analyses. β-actin was used as the loading control. Statistical analysis was performed using one-way ANOVA followed by Tukey's HSD post-hoc test for multiple comparisons (***$p < 0.001$, *$p < 0.05$). NG, normal glucose; HG, high-glucose.

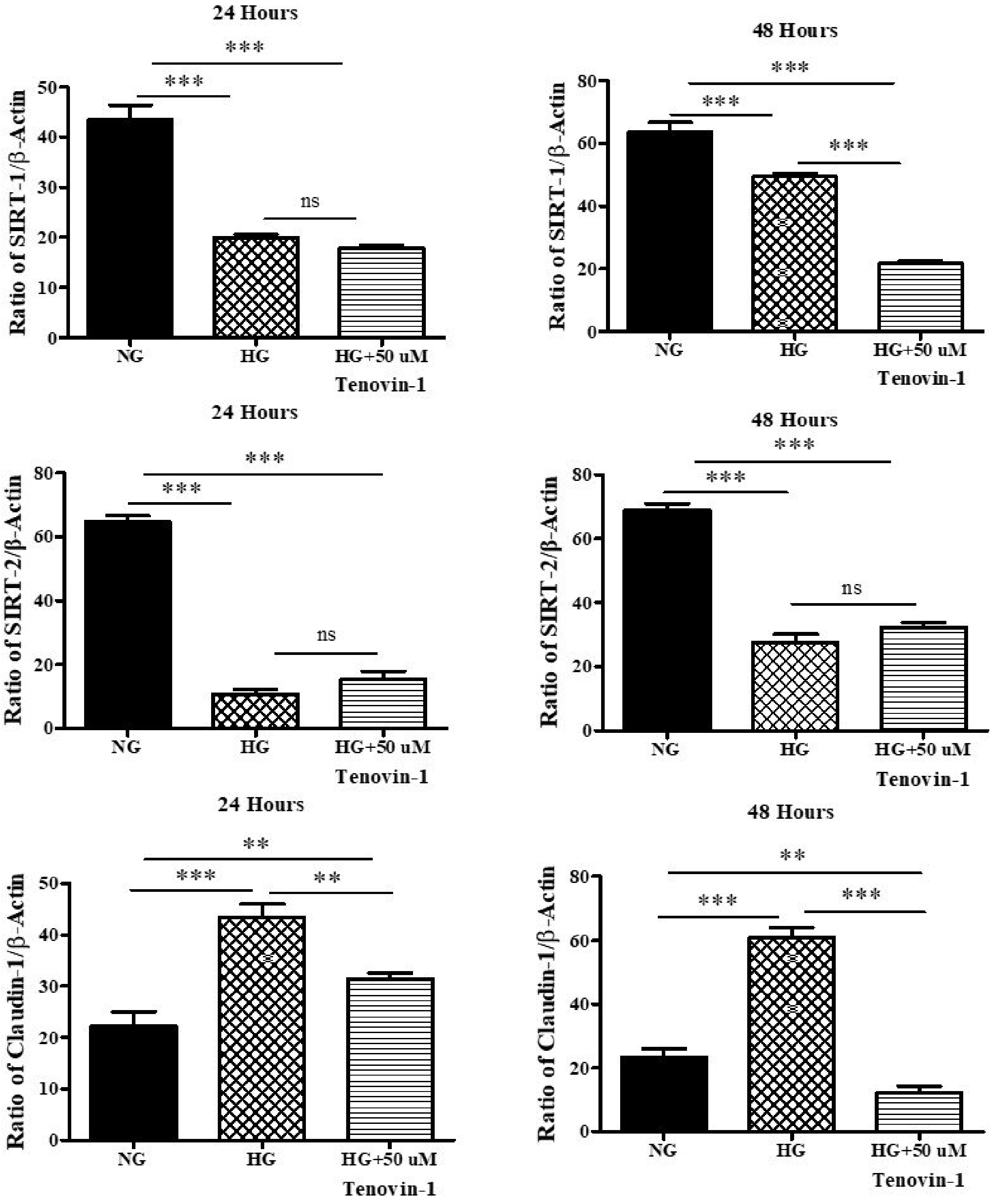

Figure S2. Band intensities of SIRTs and claudin-1 from in-vitro analysis: Band intensities of SIRTs and claudin-1 from in-vitro analysis of NRK-52E cell lysates were quantified by densitometric analysis and have been presented as relative ratio of three repetitive analyses. β-actin was used as the loading control. Statistical analysis was performed using one-way ANOVA followed by Tukey's HSD post-hoc test for multiple comparisons (***$p < 0.001$, **$p < 0.01$). NG, normal glucose; HG, high-glucose.
